# The Effect of Integrating Genomic Information into Genetic Evaluations of Chinese Merino Sheep

**DOI:** 10.3390/ani10040569

**Published:** 2020-03-28

**Authors:** Chen Wei, Hanpeng Luo, Bingru Zhao, Kechuan Tian, Xixia Huang, Yachun Wang, Xuefeng Fu, Yuezhen Tian, Jiang Di, Xinming Xu, Weiwei Wu, Hanikezi Tulafu, Maerziya Yasen, Yajun Zhang, Wensheng Zhao

**Affiliations:** 1 College of Animal Science, Xinjiang Agricultural University, Urumqi 830052, China; weichenwjf@126.com; 2 Key Laboratory of Genetics Breeding and Reproduction of Xinjiang Cashmere and Wool Sheep, Institute of Animal Science, Xinjiang Academy of Animal Science, Urumqi 830011, Chinadijing69@163.com (J.D.);; 3 Laboratory of Animal Genetics, Breeding and Reproduction, Ministry of Agriculture of China, National Engineering Laboratory of Animal Breeding, College of Animal Science and Technology, China Agricultural University, Beijing 100193, China; 4 Xinjiang Gonaisi Fine Wool Sheep-Breeding Farm, Ili Kazak Autonomous Prefecture 835800, China

**Keywords:** Chinese Merino sheep, wool traits, Bayesian inference, single-step GBLUP

## Abstract

**Simple Summary:**

Genetic improvement of wool production and quality traits in fine-wool sheep is an appealing option for enhancing the market value of wool products. We estimated genetic parameters and the accuracies of estimated breeding values for various wool production and quality traits in fine-wool sheep using pedigree-based best linear unbiased prediction (PBLUP) and single-step genomic best linear unbiased prediction (ssGBLUP) strategies. ssGBLUP performed slightly better than PBLUP for the studied traits. Therefore, the single-step genetic evaluation method could be successfully implemented in genomic evaluations of fine-wool sheep and the prediction of future breeding values in young Merino sheep as part of an early preselection strategy in the near future.

**Abstract:**

Genomic evaluations are a method for improving the accuracy of breeding value estimation. This study aimed to compare estimates of genetic parameters and the accuracy of breeding values for wool traits in Merino sheep between pedigree-based best linear unbiased prediction (PBLUP) and single-step genomic best linear unbiased prediction (ssGBLUP) using Bayesian inference. Data were collected from 28,391 yearlings of Chinese Merino sheep (classified in 1992–2018) at the Xinjiang Gonaisi Fine Wool Sheep-Breeding Farm, China. Subjectively-assessed wool traits, namely, spinning count (SC), crimp definition (CRIM), oil (OIL), and body size (BS), and objectively-measured traits, namely, fleece length (FL), greasy fleece weight (GFW), mean fiber diameter (MFD), crimp number (CN), and body weight pre-shearing (BWPS), were analyzed. The estimates of heritability for wool traits were low to moderate. The largest h^2^ values were observed for FL (0.277) and MFD (0.290) with ssGBLUP. The heritabilities estimated for wool traits with ssGBLUP were slightly higher than those obtained with PBLUP. The accuracies of breeding values were low to moderate, ranging from 0.362 to 0.573 for the whole population and from 0.318 to 0.676 for the genotyped subpopulation. The correlation between the estimated breeding values (EBVs) and genomic EBVs (GEBVs) ranged from 0.717 to 0.862 for the whole population, and the relative increase in accuracy when comparing EBVs with GEBVs ranged from 0.372% to 7.486% for these traits. However, in the genotyped population, the rank correlation between the estimates obtained with PBLUP and ssGBLUP was reduced to 0.525 to 0.769, with increases in average accuracy of 3.016% to 11.736% for the GEBVs in relation to the EBVs. Thus, genomic information could allow us to more accurately estimate the relationships between animals and improve estimates of heritability and the accuracy of breeding values by ssGBLUP.

## 1. Introduction

In China, sheep and lambs are raised mainly in large farm flocks in the northern border regions and northwestern regions and in small ranching operations in the northeast. Most of the 164,658 million sheep in China are wool or dual-purpose flocks (FAO, 2018, http://www.fao.org/faostat/en). Wool generally accounts for 20% of commercial return for wool producers. In 2017, China exported 407,000 tons of wool. However, over the last two decades, the wool clips in areas of major production have become progressively finer, while textile technology for processing superfine-wool types has improved. Consequently, the premium raw-wool prices set for fine- and superfine-wool categories have increased significantly compared with those for other types of wool. Wool quality and production traits are key features that reflect a fine-wool sheep’s economic value. Meanwhile, subjectively-assessed wool traits are widely used to select breeding ewes and rams in the sheep industry. Thus, these traits are often used together to determine the quality and grade of wool, as well as the main criteria for determining the price of wool. Breeding programs for fine-wool sheep have traditionally focused on improving wool production and wool quality. The genetic improvement of these traits is an appealing option for enhancing the market value of wool products.

Genetic evaluations of fine-wool sheep are mainly based on phenotypes and pedigree information collected with traditional quantitative genetic approaches for artificially inseminated (AI) rams based on progeny testing [1,2]. The wool traits of rams may be assessed and measured only once per year. Fine-wool sheep exhibit seasonal patterns of mating, with a low lambing percentage and limited offspring. Moreover, the assessment of wool traits requires well-trained technicians and specialized equipment, which results in costly measurements and a small amount of data. Therefore, the accuracy of genetic evaluations of wool traits in rams is very low. In addition, breeding for wool traits is hampered by difficulties associated with uncertainty in pedigree data and implementing appropriate breeding strategies. With advances in molecular technology and new biological analyses, researchers have established methods for incorporating variation at the very basic DNA level into genetic evaluations, while the availability of a high-throughput single nucleotide polymorphism (SNP) panel for sheep has created the opportunity for genomic selection (GS) [3]. Related studies have been carried out on wool [4,5,6,7], meat [8,9,10], and dairy [11,12] sheep. In New England, genomic information considerably improved the rate of genetic gain for meat and wool production in the sheep industry [13]. Meanwhile, the cumulative net present value for Australian commercial sheep increased with the implementation of genomic information, which increased the returns of genomic scenarios by 2.7-fold on average [7]. The molecular basis of wool traits in fine-wool sheep has been investigated by candidate gene [14,15,16,17] and quantitative trait locus (QTL) [18,19,20,21] detection approaches, but there have been few studies on GS.

Genomic studies of sheep have focused mostly on models, methods, and genomic estimated breeding value (GEBV) accuracy [9,22,23]. Some genomic methods have been examined and implemented, but the most comprehensive option for genomic evaluation is single-step genomic best linear unbiased prediction (ssGBLUP) [24,25]. ssGBLUP has the advantage of synchronously using the phenotypes and pedigrees of genotyped and ungenotyped animals, combined with a pedigree–genomic relationship matrix (***H***). Compared with pedigree-based best linear unbiased prediction(PBLUP) and genomic BLUP (GBLUP), ssGBLUP increases the accuracy of GEBVs in many livestock [11,26,27,28,29,30]. 

This study aims to explore the feasibility of breeding for wool traits in fine-wool sheep using genomic information. Therefore, the objective of this study was to compare estimates of genetic parameters for various wool production and quality traits in fine-wool sheep between relationship matrices. Moreover, the effects of the estimation methods on the accuracy of the EBVs were analyzed. 

## 2. Materials and Methods

### 2.1. Phenotype Data

Data from 28,391 yearlings animals (born in 1991–2017) were collected at Xinjiang Gonaisi Fine Wool Sheep-Breeding Farm (latitude: 43°03′–43°41′ N, longitude: 82°28′–84°56′ E), China. The pedigree file used for the analysis contained 41,830 animals, with each animal traced back three generations. In the full data set, one ram had a maximum of 385 offspring with data, and 573 rams had only one offspring. More than 3386 dams had 2 or more offspring with data available (156 dams had between 4 and 8 offspring). Furthermore, 663 full-sibling groups had 2 to 4 siblings. Only one shearing record was included per animal in the analysis. All animals were selected and eliminated on the basis of production performance classified from April to May each year. Shearing was carried out in June each year, and the first shearing took place at 13 to 15 months of age. The flock management of all animals was consistent with that reported by Zhao et al. [31]. 

Subjectively-assessed wool traits, namely, spinning count (SC), crimp definition (CRIM), oil (OIL), and body size (BS), and objectively-measured traits, namely, fleece length (FL), greasy fleece weight (GFW), mean fiber diameter (MFD), crimp number (CN), and body weight pre-shearing (BWPS), were analyzed. The scales used for the subjectively-assessed wool traits in Merino sheep are described in Table 1. All the samples were evaluated by persons familiar with wool production and evaluation, i.e., scientists and well-trained technicians. Each trait was assessed according to the P.R.C. GB/T 26939-2011 national standard. Before shearing, a midside wool sample (20–30 g) was taken from the left side of each animal and delivered to the Key Laboratory of Genetic Breeding and Reproduction of Xinjiang Cashmere and Wool Sheep for the measurement of MFD following the standardized methods set by the International Wool Textile Organization (IWTO) and China Fiber Inspection Bureau (CFIB). 

### 2.2. Genotype Data

A total of 965 ewes and 21 rams were genotyped with the Ovine 50K SNP BeadChip. The SNPs were obtained by common SNPs in the 50K bead chips as target files for analysis. Quality control of SNP genotypes was carried out with PLINK software [32]. All genotyped animals had a call rate greater than 0.90. An SNP was removed if the call rate was less than 0.90, the minor allele frequency (MAF) was less than 0.01, or it significantly deviated from Hardy–Weinberg equilibrium (*p* < 1 × 10^−6^). After quality control, 40,457 SNPs from 965 animals were reserved for analysis. 

### 2.3. Variance Component Estimation

Variance components were estimated by Bayesian inference using threshold models for the subjectively-assessed wool traits and linear models for the objectively-measured traits that differed in the relationship matrix used.

All wool traits were examined with the following model:

$$Y=Xb+z_{1}h+z_{2}a+e$$

where Y is a vector of SC, CRIM, OIL, BS, FL, GFW, MFD, CN, or BWPS; X is the incidence matrix for fixed effects; b is the related vector; $h\sim N\left(0,I\sigma_{h}^{2}\right)$ is the random flock effect, with ***I*** and $\sigma_{h}^{2}$ denoting the identity matrix and flock variance, respectively; $z_{1}$ is a vector of the random flock effect (156 classes); $z_{2}$ is the incidence matrix for random genetic effects and a is the related vector; and $e\sim N\left(0,I\sigma_{e}^{2}\right)$ represents the random residuals, with $\sigma_{e}^{2}$ denoting the residual variance. The fixed effects considered in the model were year (26 classes), appraiser (21 classes), dam age (8 classes), sex (2 classes), and strain (8 classes).

The additive genetic effect was modeled using two kinds of genetic (co)variance structures: for PBLUP [33], $a\sim N\left(0,A\sigma_{a}^{2}\right)$, where ***A*** and $\sigma_{a}^{2}$ denote the pedigree-based additive genetic relationship matrix and additive genetic variance, respectively, and for ssGBLUP [34], $a\sim N\left(0,H\sigma_{a}^{2}\right)$, where ***H*** denotes the pedigree-genomic relationship matrix. ***H*** was derived given ***A*** and ***G*** via the following formula:

$$H=\left[\begin{array}{cc} A_{11}-A_{12}A_{22}^{-1}A_{21}+A_{12}A_{22}^{-1}GA_{21} & A_{12}A_{22}^{-1}G\\ GA_{22}^{-1}A_{21} & G \end{array}\right]$$

where subscripts 1 and 2 of the pedigree relationship ***A*** indicate subsets of ungenotyped and genotyped animals, respectively; G is the genomic relationship matrix, calculated as $G=\frac{MM^{\prime }}{2\sum_{k=1}^{m}p_{k}\left(1-p_{k}\right)}$, where ***M*** is an incidence matrix of SNP effects, with elements $\left(0-2p_{j}\right)$, $\left(1-2p_{j}\right)$, and $\left(2-2p_{j}\right)$ denoting 11 homozygous, 12 or 21 heterozygous, and 22 homozygous, respectively; and $p_{j}$ is the MAF of the ***j*** th SNP. Thus, the inverse of the ***H*** is

$$H^{-1}=A^{-1}+\left[\begin{array}{cc} 0 & 0\\ 0 & G^{-1}+A_{22}^{-1} \end{array}\right]$$

Heritability was calculated as

$$h^{2}=\frac{\sigma_{a}^{2}}{\sigma_{a}^{2}+\sigma_{h}^{2}+\sigma_{e}^{2}}$$

GIBBS1F90 and THRGIBBS1F90 of BLUPF90 software [35] were employed to estimate the genetic parameters using the Gibbs sampling methodology for Bayesian inference. The sample number, burn-in length and sampling interval were 100,000, 10,000, and 50, respectively. Convergence of the Gibbs chain was checked by the Geweke diagnostic in the CODA package in R [36].

### 2.4. Prediction Accuracy

The accuracy of animal breeding values was calculated as

$$accuracy=\sqrt{1-SEP^{2}/\sigma_{a}^{2}}$$

where ***SEP*** is the standard error of prediction and $\sigma_{a}^{2}$ is the direct additive genetic variance.

## 3. Results

The descriptive statistics for the wool traits, including the mean, standard deviation, minimum, maximum, and coefficient of variation, are summarized in Table 2. 

### 3.1. Genetic Parameters of Wool Traits

As shown in Table A1, the converged parameter chains of additive genetic variance were used to obtain variance components of wool traits under different relationship matrices by Bayesian inference (Gibbs sampling), according to the Geweke diagnostic (the ratio between the first half and second half of the samples should be <1). The heritability estimates showed relevant variation in different wool traits and matrices (Table 3). Overall, the estimates of heritability for wool traits were low to moderate. The largest h^2^ values were observed for FL (0.277) and MFD (0.290) with ssGBLUP. The smallest estimates were obtained for OIL (0.070 and 0.072) with both prediction models. Moreover, the heritability estimates of objectively-measured traits were moderate, except that for CN (0.089), which was lower with PBLUP. The heritabilities estimated for wool traits with ssGBLUP were slightly higher than those obtained with PBLUP. Furthermore, the ssGBLUP and PBLUP estimates of GFW were very similar. For all models, the standard errors of heritability estimates were low (between 0.013 and 0.071), as expected for such a small data set. Surprisingly, little improvement occurred in terms of the accuracy of the estimated heritability, as the standard errors were similar between models with the ***A*** or ***H***. 

### 3.2. Accuracy of EBV and GEBV Predictions

**Figure A1:**
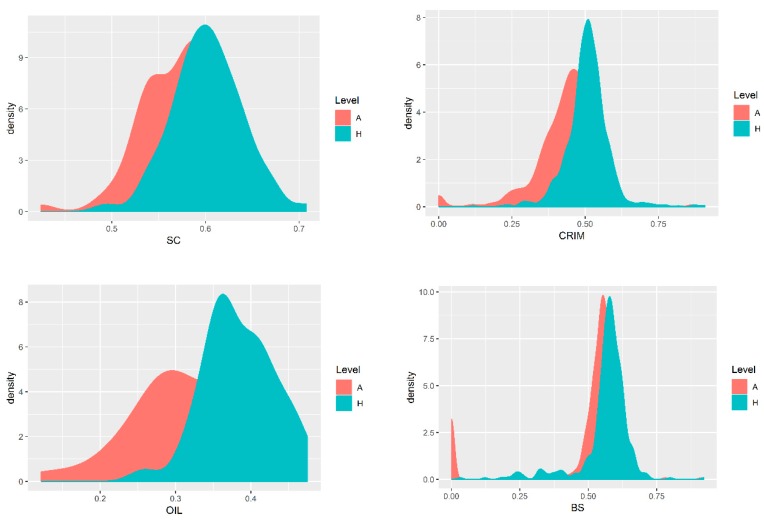

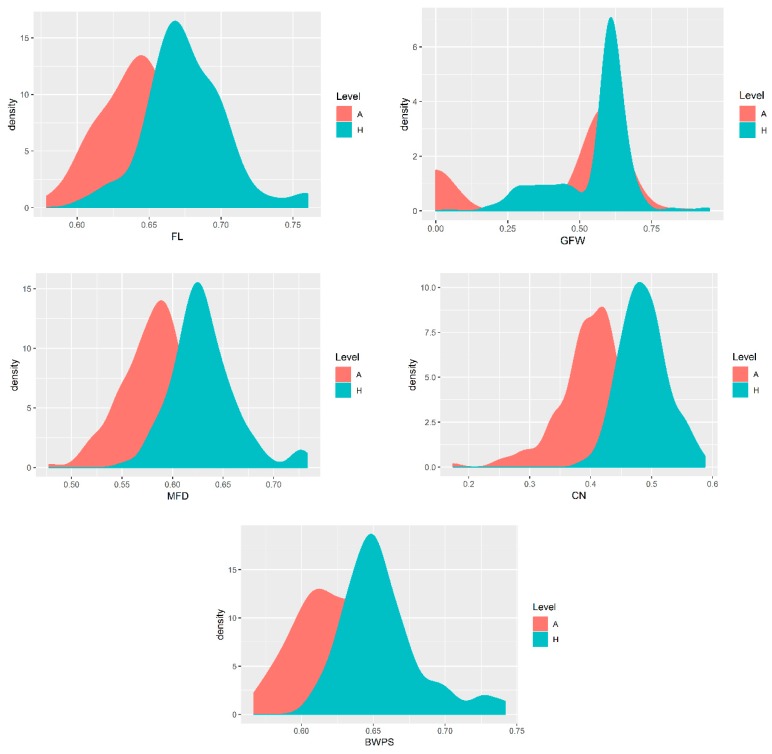
Density map of breeding value accuracy for different traits in genotyped animals under different matrices: pedigree-based approach (A, PBLUP) and combined pedigree and genomic information-based approach (H, ssGBLUP).

The data in Table 4 show that the accuracies of breeding values were low to moderate, ranging from 0.362 (CN) to 0.573 (BWPS) and from 0.318 (OIL) to 0.676 (FL) in the whole population and genotyped subpopulation, respectively. BWPS showed the highest accuracy for the different prediction models, in both the whole population (0.573) and the genotyped subpopulation (0.656). The highest GEBV accuracy was observed for FL (0.676) in the genotyped subpopulation. OIL showed the lowest accuracy of prediction with both methods. Simultaneously, the correlation between EBVs and GEBVs ranged from 0.717 to 0.862 for the whole population, and the increase in accuracy for EBVs compared with GEBVs ranged from 0.372% to 7.486% for these traits. However, for only the genotyped animals, the correlation between the estimates obtained with PBLUP and ssGBLUP was reduced to 0.525 to 0.769, with increases of 3.016% to 11.736% in the average accuracy of the GEBVs compared with that of the EBVs. Figure A1 shows the frequency distribution of EBV and GEBV accuracies for genotyped animals when using different matrices. The resulting distribution for the ***H*** of wool traits was skewed to the right compared with that of the ***A***, indicating that the ***H*** improved the accuracy of GEBV predictions. Additionally, the figure shows that the number of individual animals with ***A*** estimated EBV accuracies equal to 0 for SC, CRIM, OIL, FL, GFW, and BWPS was larger than that with ***H*** estimated GEBV accuracies equal to 0 for these traits. Therefore, the ***H*** can be used to obtain accurate GEBVs for such individuals via genomic relationship information. 

## 4. Discussion

### 4.1. Genetic Parameters of Wool Traits

The heritability estimates based on pedigree models for the objectively-measured traits were inconsistent with those reported in a previous study using a similar data set [1]. Compared with the results of previous studies, the pedigree-based h^2^ values for GFW and BWPS in the present study were higher, but the heritabilities of MFD and FL were not different. In contrast, the genomic information-based h^2^ values for MFD and BWPS were larger than the pedigree-based ones. Thus, the gene frequency in the fine-wool sheep (Xinjiang type) population has changed over 10 years of breeding. Moreover, many investigations have used the restricted maximum likelihood (REML) method and reported high heritability estimates for GFW, MFD, FL, and BWPS in Merino sheep or dual-purpose breeds, ranging from 0.37 to 0.74 [37,38,39,40]. Meanwhile, the heritability estimates for subjectively-assessed wool traits were low to medium and ranged from 0.070 to 0.204 when using threshold models for Bayesian inference in the present study. SC is a very important economic trait in fine-wool sheep, and the heritability estimate for this trait was 0.201 ± 0.046 in this study. This finding is inconsistent with that of Hanford et al. [41,42,43], who reported heritability of SC in three different fine-wool sheep populations of 0.16 ± 0.06, 0.41 ± 0.05, and 0.37 ± 0.07 when using REML. Furthermore, the standard error of heritability for SC was lower in the present study. The present heritability estimates for CRIM and OIL were lower than those reported by Matebesiet al. [44,45,46]. Nevertheless, the heritability estimates for CRIM and OIL based on pedigree or genomic information were lower, indicating that subjectively-assessed wool traits are greatly influenced by environmental factors. For example, grazing lambs on pasture in winter led to malnutrition caused by poor feeding and management conditions, cold weather, and other factors, facilitating wool knotting and quality problems [47]. However, many factors that may lead to bias in heritability estimates of traits, such as data structure (i.e., relationships between phenotypically-similar individuals and the number of individuals with recorded phenotypes), pedigree structure (i.e., depth of the pedigree, full-sibling number, and half-sibling number), trait heritability, and parentage errors in the registration system. Therefore, there was also considerable group-level genetic variation in these traits, regardless of the approach or models used, breed and type of animal, and environmental and management conditions, and the results were generally not consistent [38]. 

The current study revealed that the heritability estimates of wool traits obtained using genomic information were slightly higher than those obtained with a pedigree, whereas the standard errors of the heritability estimates were generally equivalent. In particular, differences in h^2^ estimates obtained with genomic methods mainly increased the additive genetic variance. However, there are several potential explanations for the difference in estimated genetic variances between the models based on pedigree and genomic relationships. First, the ***A*** and ***H*** were different, displaying a difference in scale for the diagonal elements, especially considering the information on the Mendelian sampling component in the ***H***. Veerkamp et al. [48] demonstrated that rescaling ***H*** according to the eigenvalues of ***A*** slightly changed the genetic variances. Meanwhile, Forni et al. [49] reported that parameter estimates may be biased when the genomic relationship coefficients are on a different scale than the pedigree-based coefficients. This occurs because of fundamental differences between the genetic models of the ***A*** and ***H*** [50]. A second reason is the genetic structure of sheep populations. Swan et al. [8] estimated genetic parameters for carcass traits in a multibreed sheep population using phenotypic, pedigree, and genomic information. As a result, for traits with little change in heritability between methods, the accuracies of heritability estimates were similar, consistent with the results of this study. Contrary to what was observed in the present study, Veerkamp et al. [48] reported that estimates of genetic variance for milk yield, dry matter intake, and body weight were lower when calculated based on genomic relationships than when using pedigree relationships in a dairy cattle sample of comparable size, but the standard errors indicated that using the genomic relationships provided more accurate estimates of heritability, and the results were generally consistent with those of Haile et al. [51] and Loberg et al. [52]. In addition, Kijas et al. [53] found that the genetic structure of sheep populations was characterized by less short-range linkage disequilibrium than that of cattle populations. A third reason may be a serious lack of parental IDs in the flock registration system and a high pedigree error rate. On the one hand, the ear tags of grazing sheep can be easily missed. On the other hand, the appraiser may make an error when registering the sheep. Therefore, the accuracy of sheep pedigrees is often questionable due to the high frequency of unknown parents. Although the results of the current study are promising and combining data from genotyped and non-genotyped animals appears to be worthwhile, in some cases, using genotyped animals may be preferable over collecting more data. 

### 4.2. Accuracy of EBV and GEBV Predictions

In this study, the breeding value accuracies for wool traits were low to moderate. In general, the relative increase in accuracy for PBLUP compared to ssGBLUP was larger for genotyped animals than for the whole population. However, there were many reasons for the above results. First, the reference population in this study consisted mainly of females (less than 3% of the genotyped animals were rams), consistent with the conditions of by Molina et al. [28]. However, rams must be genotyped, which is important not only in breeding, but also in improving accuracy at the herd level. Second, our genotyped population was small (only 4% of the animals were genotyped), and the distribution of animal genotypes was not homogeneous. Importantly, with increases in reference population size and pedigree data completeness, the accuracy of EBVs or GEBVs will increase [54]. In addition, Li et al. [55] reported that the accuracies of GEBVs for the ssGBLUP method were greater for traits with a larger reference population (numbers of animals phenotyped and genotyped) and for traits with higher heritabilities. Similar studies in other sheep populations have also reported a strong advantage of GEBVs in the evaluation of wool-related traits. For example, using genomic data from 7180 Merino sheep, Daetwyler et al. [4] obtained GEBV accuracies for MFD and GFW (yearlings and adults) ranging from 0.23 to 0.79 by GBLUP and BayesA methods. Meanwhile, Moghaddar et al. [6] obtained GEBV accuracies of 0.33 to 0.75 for yearling wool traits by GBLUP based on genomic data from approximately 5000 Merino sheep, and the accuracies were even higher for GFW and MFD (0.75 and 0.72). However, the reasons for the high accuracies in the above studies on wool traits were the large proportions of genotyped animals as well as the high heritabilities of yearling and adult wool traits. There are few reports on the genomic evaluation of subjectively-assessed wool traits, mainly because these traits are categorical or threshold traits and generally characterized by low selection efficiency. However, in this study, the ssGBLUP method slightly improved the accuracy of CRIM and OIL GEBVs. A third reason may be incomplete genotype datasets with errors due to the use of different SNP panels in sheep populations. In the New Zealand sheep industry, Ventura et al. [56] found that the use of different SNP panels and selection of animals for genotyping using both high- and low-density SNP panels impacted imputation accuracy in genomic evaluation. At the same time, Nordbø et al. [57] proved that erroneous SNP data or incomplete imputation of missing SNPs explained the difference between non-genotyped and genotyped animals examined with different SNP panels. 

Additionally, Swan et al. [5] reported that the accuracies of GEBVs for yearling wool traits obtained by the PBLUP method were slightly higher than those obtained with the GBLUP method in young Merino rams. As a result, the authors argued that a single-step analysis combining a genomic relationship matrix with a standard pedigree-based relationship matrix should be incorporated into routine evaluations in the near future. However, Brown et al. [58] reported encouraging improvements in accuracy for meat and reproduction traits in Australian sheep when using ssGBLUP. Several studies of different traits and livestock species have concluded that single-step genomic evaluations provide more accurate predictions of genetic merit than does the classical PBLUP method [11,29,59,60,61].

## 5. Conclusions

Genomic evaluation in Merino sheep using ssGBLUP is feasible for both continuous and categorical traits. The first Merino sheep genomic evaluation carried out in China has shown that it is possible to improve heritability and breeding value accuracies for wool traits (albeit modestly) by the ssGBLUP method in a sample of limited size. Our findings indicated that the estimated heritability and breeding value accuracies for wool traits were low to medium when using PBLUP and ssGBLUP. However, the estimated parameters provide a basis for calculating more accurate Merino sheep breeding values. It is likely that a method of this type will be implemented in fine-wool sheep genetic evaluations in the near future. The next challenge is to develop improved single-step analyses that can continue to accommodate larger numbers of genotyped animals in the future. Therefore, if the population of reference were increased beyond the size used in this study (especially the proportion of rams), the results would allow improving the breeding value accuracy for wool traits and the prediction of future breeding values of young Merino sheep as part of an early preselection strategy in the near future. 

## Figures and Tables

**Table 1:** Scale used for the subjectively-assessed wool traits in Merino sheep.

Trait ^1^	Scale of Assessment				
	1	2	3	4	5
SC	poor	below average	average	above average	excellent
CRIM	poor	below average	average	above average	excellent
OIL	dark yellow	faint yellow	milk white	white	-
BS	narrow	below average	average	above average	wide

^1^ SC: spinning count; CRIM: crimp definition; OIL: oil; BS: body size.

**Table 2:** Description of wool traits in Chinese Merino sheep (Xingjiang type).

	Traits ^1^	Number	Minimum	Maximum	Average	SD	CV
Subjectively-assessed wool traits	SC	28390	1.00	5.00	2.78	0.84	0.30
	CRIM	19356	1.00	5.00	3.83	0.82	0.21
	OIL	21173	1.00	4.00	3.36	0.78	0.23
	BS	27110	1.00	5.00	2.80	1.22	0.44
Objectively-measured traits	FL/cm	28658	6.00	15.00	10.12	1.10	0.11
	GFW/kg	27379	1.00	9.00	3.98	0.94	0.24
	MFD/μm	5140	12.77	28.13	18.41	2.28	0.12
	CN/per 2.5 cm	4326	7.00	25.00	12.50	2.53	0.20
	BWPS/kg	25676	20.00	78.00	34.30	5.47	0.16

^1^ SC: spinning count; CRIM: crimp definition; OIL: oil; BS: body size; FL: fleece length; GFW: greasy fleece weight; MFD: mean fiber diameter; CN: crimp number; BWPS: body weight pre-shearing; SD: standard deviation; CV: coefficient of variation.

**Table 3:** Variance components and heritability of wool traits obtained using the pedigree relationship matrix (pedigree-based best linear unbiased prediction (PBLUP)) and combined genomic-pedigree matrix (single-step genomic best linear unbiased prediction (ssGBLUP)) (SE of variance components and heritability reported in parentheses).

	PBLUP				(ssGBLUP)			
Traits ^1^	$\sigma_{a}^{2}$	$\sigma_{h}^{2}$	$\sigma_{e}^{2}$	$h^{2}$	$\sigma_{a}^{2}$	$\sigma_{h}^{2}$	$\sigma_{e}^{2}$	$h^{2}$
SC	0.011 (0.002)	0.014 (0.006)	0.029 (0.014)	0.201 (0.046)	0.011 (0.002)	0.014 (0.006)	0.029 (0.013)	0.204 (0.046)
CRIM	0.370 (0.340)	1.523 (0.901)	2.796 (1.86)	0.079 (0.071)	0.463 (0.376)	1.619 (0.864)	2.934 (1.761)	0.107 (0.070)
OIL	0.196 (0.086)	1.366 (0.077)	1.245 (1.364)	0.070 (0.037)	0.210 (0.088)	1.418 (0.786)	1.297 (1.431)	0.072 (0.039)
BS	0.183 (0.028)	0.350 (0.153)	0.593 (0.391)	0.163 (0.049)	0.179 (0.028)	0.312 (0.153)	0.597 (0.373)	0.165 (0.049)
FL	0.302 (0.016)	0.228 (0.029)	0.579 (0.014)	0.272 (0.016)	0.308 (0.016)	0.227 (0.029)	0.578 (0.014)	0.277 (0.016)
GFW	0.123 (0.008)	0.245 (0.031)	0.343 (0.007)	0.173 (0.013)	0.123 (0.008)	0.246 (0.003)	0.343 (0.007)	0.173 (0.013)
MFD	0.827 (0.127)	0.309 (0.080)	2.408 (0.117)	0.233 (0.035)	1.024 (0.114)	0.302 (0.076)	2.201 (0.101)	0.290 (0.031)
CN	0.506 (0.167)	0.276 (0.083)	4.880 (0.182)	0.089 (0.029)	0.859 (0.191)	0.255 (0.078)	4.542 (0.197)	0.152 (0.032)
BWPS	5.767 (0.346)	8.064 (1.072)	12.189 (0.298)	0.222 (0.015)	5.790 (0.336)	8.009 (1.052)	12.166 (0.284)	0.223 (0.015)

^1^ SC: spinning count; CRIM: crimp definition; OIL: oil; BS: body size; FL: fleece length; GFW: greasy fleece weight; MFD: mean fiber diameter; CN: crimp number; BWPS: body weight pre-shearing; $\sigma_{a}^{2}$: additive genetic variance; $\sigma_{h}^{2}$: flock effect variance; $\sigma_{e}^{2}$: residual variance; h^2^: heritability; SE: standard error.

**Table 4:** Comparison of estimated breeding value (EBV) and genomic estimated breeding value (GEBV) accuracies (PBLUP and ssGBLUP) and increases in accuracy (Δacc) obtained for the whole population and the genotyped subpopulation of Chinese Merino sheep.

	Whole Population				Genotyped Subpopulation			
Traits ^1^	PBLUP	ssGBLUP	Δacc (%)	Correlation	PBLUP	ssGBLUP	Δacc (%)	Correlation
SC	0.532	0.539	0.699	0.855 **	0.569	0.602	3.274	0.769 **
CRIM	0.367	0.433	6.638	0.767 **	0.430	0.507	7.712	0.664 **
OIL	0.389	0.414	2.518	0.777 **	0.318	0.384	6.659	0.682 **
BS	0.517	0.527	0.993	0.852 **	0.503	0.563	6.032	0.752 **
FL	0.559	0.563	0.372	0.862 **	0.646	0.676	3.016	0.668 **
GFW	0.517	0.523	0.595	0.854 **	0.436	0.553	11.736	0.705 **
MFD	0.476	0.521	4.544	0.833 **	0.580	0.630	5.012	0.580 **
CN	0.362	0.437	7.486	0.717 **	0.400	0.487	8.659	0.525 **
BWPS	0.562	0.573	1.089	0.858 **	0.623	0.656	3.237	0.598 **

^1^ SC: spinning count; CRIM: crimp definition; OIL: oil; BS: body size; FL: fleece length; GFW: greasy fleece weight; MFD: mean fiber diameter; CN: crimp number; BWPS: body weight pre-shearing. ** indicates correlation being significantly different from 0 (*P* < 0.01).

## Appendix A

**Table A1:** Geweke diagnostic results of the convergence of Gibbs chains for wool traits using the pedigree relationship matrix (PBLUP) and combined genomic–pedigree matrix (ssGBLUP).

Traits ^1^	PBLUP	ssGBLUP
SC	−0.03	−0.12
CRIM	−0.04	0.25
OIL	−0.14	−0.12
BS	−0.14	0.04
FL	0.02	0.05
GFW	−0.01	0.27
MFD	0.00	0.04
CN	−0.19	0.17
BWPS	−0.14	0.00

^1^ SC: spinning count; CRIM: crimp definition; OIL: oil; BS: body size; FL: fleece length; GFW: greasy fleece weight; MFD: mean fiber diameter; CN: crimp number; BWPS: body weight pre-shearing.
